# Checklist of marine macroalgae in two contiguous Marine Protected Areas in the south-western Atlantic

**DOI:** 10.3897/BDJ.12.e122350

**Published:** 2024-07-23

**Authors:** Claudia S Karez, Ricardo G Bahia, José M C Nunes, Gabriel N Santos, Rodrigo L Moura, Paulo S Salomon, Clarice C M Ribeiro, Carolina C Silva, Pedro Cardial, Gabriella A Leal, Manoela B Lyra, Leonardo T Salgado

**Affiliations:** 1 Instituto de Pesquisa Jardim Botânico do Rio de Janeiro, Rio de Janeiro, Brazil Instituto de Pesquisa Jardim Botânico do Rio de Janeiro Rio de Janeiro Brazil; 2 Laboratório de Algas Marinhas - LAMAR, Instituto de Biologia, Universidade Federal da Bahia, Salvador, Brazil Laboratório de Algas Marinhas - LAMAR, Instituto de Biologia, Universidade Federal da Bahia Salvador Brazil; 3 Instituto de Biologia & SAGE/COPPE, Rio de Janeiro, Brazil Instituto de Biologia & SAGE/COPPE Rio de Janeiro Brazil; 4 Instituto de Biologia and Núcleo Professor Rogério Vale de Produção Sustentável -SAGE/COPPE, Universidade Federal do Rio de Janeiro, Rio de Janeiro, Brazil Instituto de Biologia and Núcleo Professor Rogério Vale de Produção Sustentável -SAGE/COPPE, Universidade Federal do Rio de Janeiro Rio de Janeiro Brazil; 5 Rio de Janeiro Botanical Garden, Rio de Janeiro, Brazil Rio de Janeiro Botanical Garden Rio de Janeiro Brazil

**Keywords:** algae, south-western Atlantic Ocean, Costa das Algas Environmental Protection Area, Santa Cruz Wildlife Refuge

## Abstract

**Background:**

The Costa das Algas Environmental Protection Area (EPA) and the Santa Cruz Wildlife Refuge (WR), located in the Espírito Santo Continental Shelf, Brazil, are outstanding marine protected areas due to their high biodiversity, particularly of macroalgae. Together, these two relatively small protected areas (1,150 and 177 km^2^, respectively) harbour about a quarter of all macroalgal species recorded in Brazil.

The checklist presented herein updates the algal flora of these two protected areas with data obtained until 2019. Two hundred and sixty-five macroalgal taxa were recorded, most of which with vouchers. Checklists based on the collections of each protected area were published on: "Catálogo de Plantas das Unidades de Conservação do Brasil" (https://catalogo-ucs-brasil.jbrj.gov.br/).

**New information:**

Besides specimens collected between 2018 and 2019, the algal flora presented herein includes previous records from different Brazilian herbaria (e.g., SP, SPF, ALCB). Herbaria records may include species that do not occur in intertidal reefs (e.g., *Laminaria*). Overall, 249 macroalgal taxa and one marine angiosperm were recorded in the Costa das Algas EPA (87 new records) and 136 macroalgal taxa and one marine angiosperm in the Santa Cruz WR (46 new records). All taxa are native to Brazil and nine are endemic to Brazil. Our results provide a taxonomic foundation to support management, long-term monitoring and conservation in these protected areas.

## Introduction

In coastal zones, benthic macroalgae are important primary producers that provide habitat and food for several organisms and multiple benefits for people. They contribute for food, fisheries support, nutrient cycling, coastal protection, water quality and carbon storage (Duffy et al. 2019). However, they are often threatened by several human activities, such as coastal development, urban wastes, invasive species, climate change, oil and gas and industrial activities (Duffy et al. 2019, Hanley et al. 2024).

The Espírito Santo State (ES) encompasses one of Brazil's highest species richness of marine macroalgae, with records of about 400 species (Guimarães 2003, Flora e Funga do Brasil 2023). Its shoreline is composed of beaches, bays, inlets, mangroves, estuaries and lateritic reefs, while its continental shelf harbours a variety of sedimented bottom and rhodolith beds (Bastos et al. 2015, Quaresma et al. 2015), both punctuated by biogenic reefs and other mesoscale benthic features (Holz et al. 2020, Oliveira et al. 2023). The diversity of structurally complex hard bottom habitats contributes to the great intertidal and subtidal diversity of algae (Guimarães 2003, Amado-Filho et al. 2010, Holz et al. 2020). Moreover, the study region is situated in a transitional zone between the tropical and warm temperate phycogeographical provinces (Horta et al. 2001), allowing for the co-occurrence of species with affinities for both warm and cold waters (Horta et al. 2001).

The multiple-use Costa das Algas EPA and the no-take Santa Cruz WR are contiguous and form part of UNESCO's Mata Atlântica Biosphere Reserve. Established in 2010 by Brazil’s Federal Government, these marine protected areas (MPAs) aim at the protection of marine biodiversity and management of artisanal fisheries, and were important milestones for the protection of rhodolith beds against large-scale exploitation for the production of agriculture fertilisers [IBAMA (Instituto Brasileiro do Meio Ambiente e dos Recursos Naturais Renováveis) 2006]. Both areas have great ecological and socioeconomic importance and protect critical habitats that ensure coastal zone protection against erosion and the sustainability of artisanal fisheries, the latter being a major economic activity in the region [ICMBio (Instituto Chico Mendes de Conservação da Biodiversidade) 2023a]. Moreover, they are major tourism destinations, especially during the summer. These areas are faced with several threats, including the contamination by heavy metals from mining wastes transported by the Doce river, with highly increased concerns after the collapse of the Fundão Dam in November 2015 [ICMBio (Instituto Chico Mendes de Conservação da Biodiversidade) 2023b].

Oliveira-Filho (1977) was the first to highlight the outstanding algal diversity in ES. However, inventories are limited to a few publications (Oliveira-Filho 1969, Oliveira-Filho 1976, Oliveira-Filho 1976, Guimarães 2006, Scherner et al. 2013) and some data was only available in grey literature (Crispino 2000, Barata 2004, Carvalho 2013). Furthermore, most inventories were carried out before 2010 and did not include geographic coordinates or vouchers. In an effort to reduce this knowledge gap, this study aimed at providing an updated checklist of macroalgae for the EPA and the WR.

### Project description

**Title**: This inventory was carried out within the scope of two projects: 1) T1) Aquatic Biodiversity Monitoring Program (PMBA-FEST, RENOVA); 2) Long-Term Ecological Research Program (PELD-Abrolhos/CNPq);

**Personnel**: The research is Cláudia S Karez Post-Doctoral/CNPq project in the Jardim Botânico do Rio de Janeiro (JBRJ), supervised by Leonardo T Salgado. Identifications were made under the supervision of José Marcos C Nunes (Universidade Federal da Bahia) and Ricardo G Bahia (JBRJ) for crustose coralline algae. Both project are coordinated by Rodrigo L Moura (Universidade Federal do Rio de Janeiro).

**Study area description**: Sampling was carried out in the Espirito Santo Continental Shelf (ESCS, Oliveira et al. (2020)), south-eastern Brazil, in intertidal reefs within two contiguous marine protected areas, the Costa das Algas EPA (multiple-use) and the Santa Cruz WR (no-take).

**Funding**: The inventory was funded by the Aquatic Biodiversity Monitoring Program (PMBA-FEST, RENOVA) and the Long-Term Ecological Research Program (PELD-Abrolhos/ CNPq). Brazil's Conselho Nacional de Desenvolvimento Científico e Tecnológico (CNPq) has granted Research Productivity (JMCN, RLM, PSS, LTS) and Post-Doctoral (CSK) fellowships. We thank the “Núcleo de Gestão Integrada de Santa Cruz” - Instituto Chico Mendes de Conservação da Biodiversidade (ICMBio) for research permits and background information.

## Materials and methods

### Study areas

This study focused on macroalgae associated with intertidal reefs in Costa das Algas EPA and Santa Cruz WR, in the Espirito Santo Continental Shelf (ESCS, Oliveira et al. (2020)), south-eastern Brazil. The framework of these reefs, which dwell under a micro-tidal regime and strong fluvial influence, is composed of lateritic rocks interspaced with calcareous formations (Mazzucco et al. 2020). On the Espírito Santo coast, temperatures range from 25 to 28ºC in the summer and from 22.5 to 24.5ºC in the winter (Instituto Estadual de Meio Ambiente e Recursos Hídricos 2014). Upwelling associated to the intrusion of the South Atlantic Central Water impacts the ESCS, lowering the temperature during short periods between October and March (Guimarães 2003).

### Sampling methods

**Figure 1.**
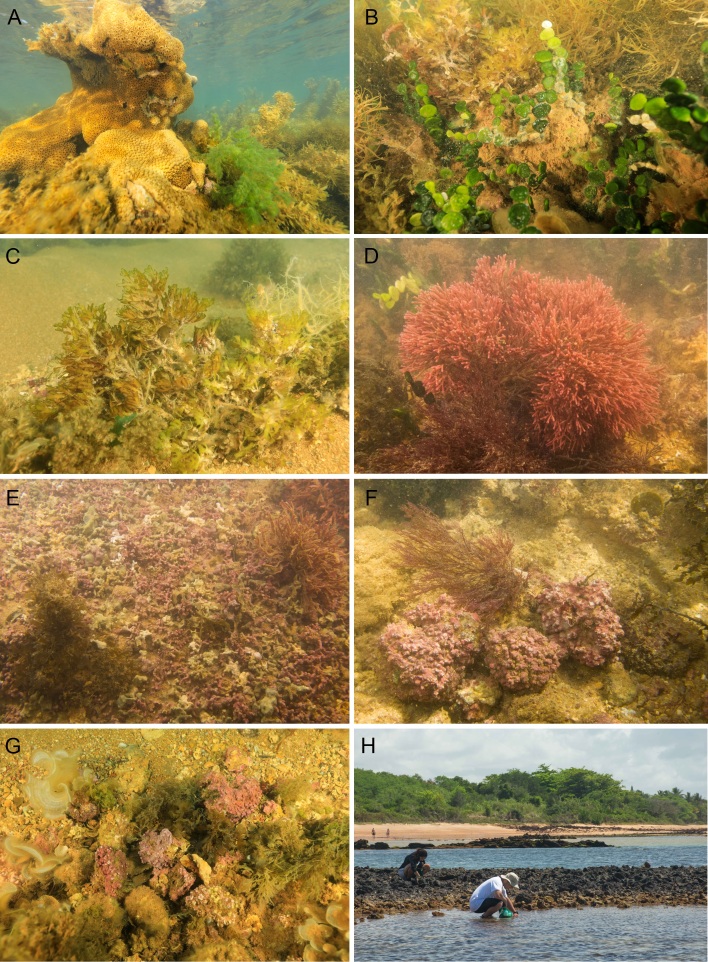
Shallow reef benthic assemblages adjacent to the coastline dominated by algae in the Costa das Algas EPA and Santa Cruz WR (2018-2019). **A** Zoanthid (*Palythoa* sp.) and algae; **B** *Halimeda* sp.; **C** *Vidaliaobtusilosa*; **D** *Tricleocarpacylindrica*; **Е** Maerls formed by living mono-specific CCA covering the sea bed associated with *Dictyoperisdelicatula*; **F** Living rhodoliths surrounded by nodules covered by turf; **G** Living rhodoliths associated with *Padina* sp. and *Sargassum* sp.; **H** Collection of macroalgae in the field; Photos: (A-C) A. Bertoncini; Photos (D-H) F. Moraes.

**Figure 2.**
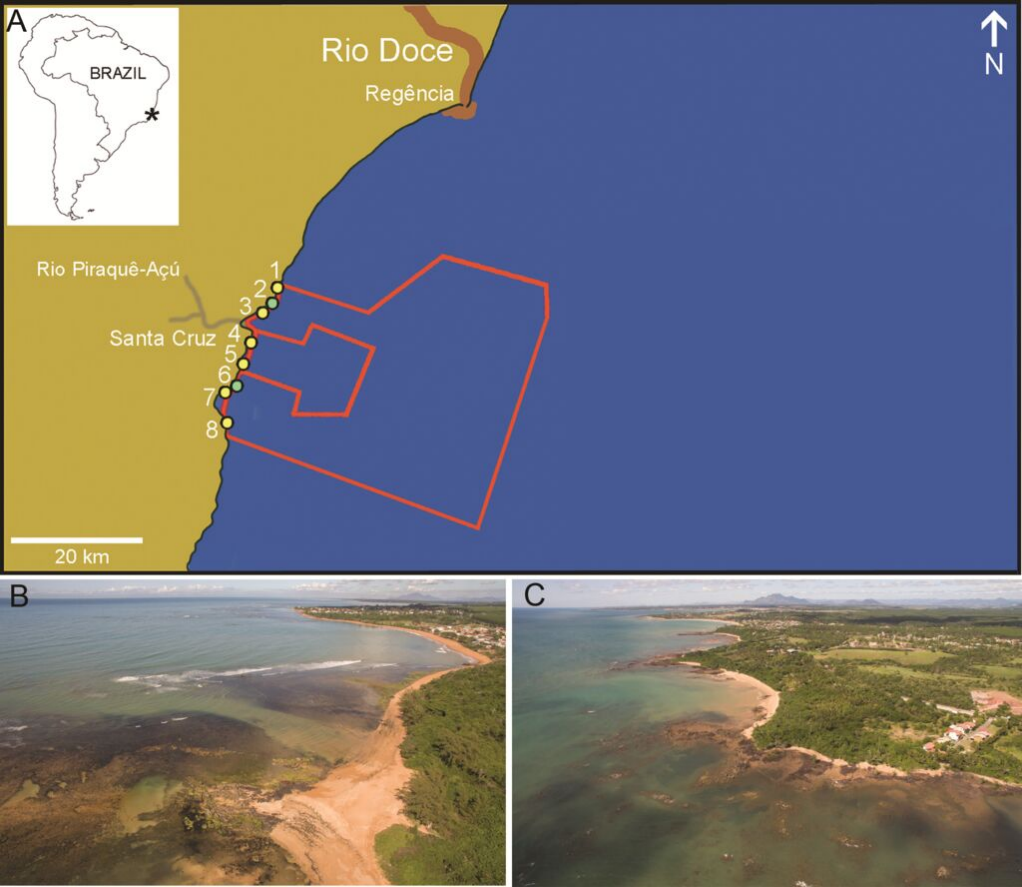
Site location and views of the two studied MPAs. **A** Sampling sites in the multiple use Costa das Algas Environmental Protection Area (larger polygon) and in the no-take Santa Cruz Wildlife Reserve (smaller polygon); **B** Costa das Algas EPA; **C** Santa Cruz WR. Photos: F. Moraes.

**Description**: The checklist was primarily based on four field surveys carried out in 2018 and 2019 at eight sites in the intertidal zone (Figs 1, 2), in the two protected areas, covering the four seasons. In addition to field surveys, previous records were obtained from databases: Jabot ("Jardim Botânico do Rio de Janeiro", JBRJ, http://www.jbrj.gov.br/jabot), Reflora ("Herbário Virtual Reflora", https://reflora.jbrj.gov.br/reflora/herbarioVirtual/) and speciesLink ("Centro de Referência em Informação Ambiental”, CRIA, https://specieslink.net/search/). Searches were based on the three municipalities "Aracruz", "Fundão" and "Serra" and only records that specified localities within protected areas' perimeters were selected. Species' names were updated following Guiry and Guiry (2023).

Checklists of species with vouchers in the main national herbaria were published on the "Catálogo de Plantas das Unidades de Conservação do Brasil" (https://catalogo-ucs-brasil.jbrj.gov.br/) for both MPAs. Here, we report 30 additional species recorded in our field surveys, but not found in the herbaria. Their abundance was assessed according to the frequencies of occurrence in the eight studied sites and four different seasons in the field surveys (2018-2019), as follows: very common (VC = 28-32 occurrences); common (C = 21-27 occurrences); frequent (F = 3-10 occurrences) and rare (R = 1 or 2 occurrences). Coralline algae were often identified only at the genus level, since species level identification of these algae usually requires molecular analysis.

**Quality control**: Identifications were based on AlgaeBase (Guiry and Guiry 2023), identification guides, regional and local floristic studies and keys (Oliveira-Filho 1977, Amado-Filho and Yoneshigue-Valentin 1990, Nunes 1998, Nunes 2005, Guimarães 2006, Figueredo and Tâmega 2007, Pacheco 2011, Bahia 2014, Ximenes et al. 2017, Santos et al. 2020 , Coutinho et al. 2022, Wynne 2022) and validated by taxonomists.

**Statistical analysis**: Diversity of macroalgae of both MPAs was summarised at family and genus level, as well as endemism and status as native or non-native, based on Flora e Funga do Brasil (2023).

## Data resources

### Geographic coverage

**Description**: The Costa das Algas EPA covers 1,150 km^2^, with about 96% of its area in the marine environment and the remaining in restingas, beaches and mangroves (Fig. 2). The Santa Cruz WR covers 177 km^2^, 98% in the marine environment. Altogether, these MPAs span about 30 km of shoreline.

**Coordinates**: Costa das Algas EPA, 20°2’17.42”S, 39°55’2.77”W, in irregular polygon; Santa Cruz WR, 20°0’32.80”S, 40°3’31.49”W, in irregular polygon.

## Checklists

### Checklist of marine macroalgae and seagrasses of Costa das Algas EPA and Santa Cruz WR

#### Chlorophyta/Ochrophyta/Rhodophyta/Tracheophyta

B477866E-80F6-5AAC-98EF-6CB5C8D3FD47

##### Notes

Macroalgae recorded in the studied areas are presented in Table 1

## Analysis

### Taxonomy coverage

**Figure 3.**
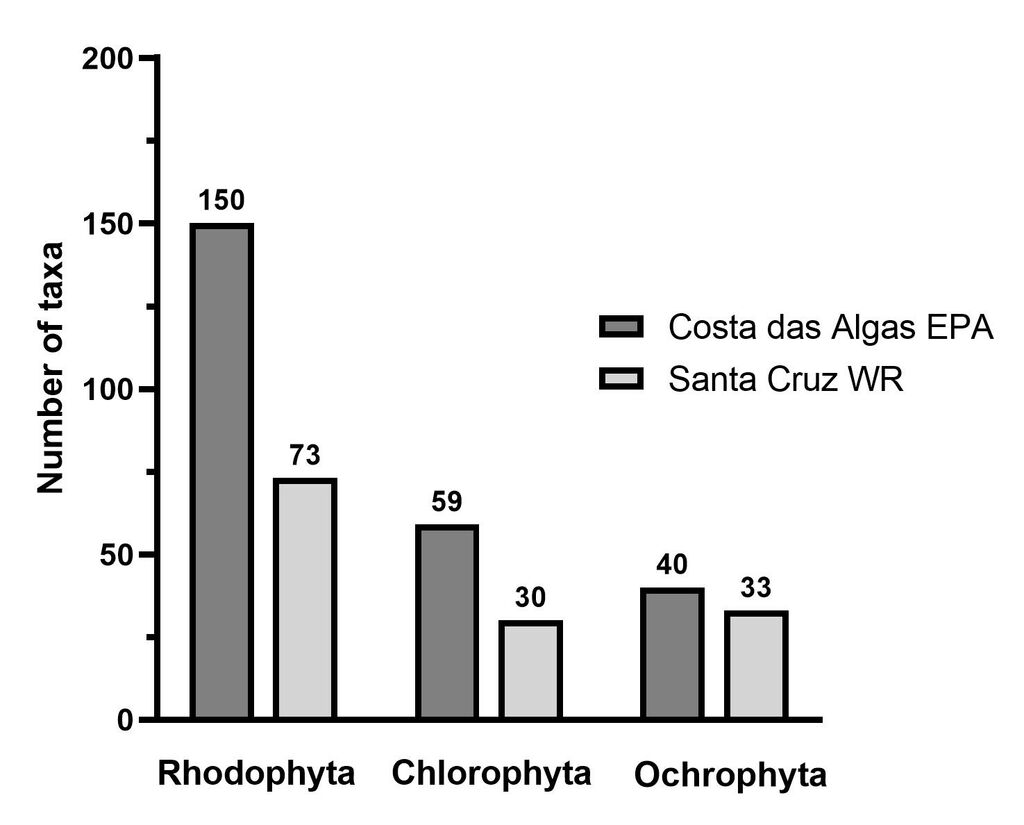
Number of macroalgal taxa of Rhodophyta, Chlorophyta and Ochrophyta in the Costa das Algas EPA and the Santa Cruz WR (ES).

Two hundred and sixty-five macroalgal taxa were recorded in the protected areas, including four varieties, one subspecies and one marine angiosperm (Table 1, Suppl. material 1, Suppl. material 2). Costa das Algas EPA (250 taxa) was richer than the Santa Cruz WR (137 taxa) due to the combination of lower sampling effort (only two sampling sites) and smaller area of the latter (Fig. 3).

Records of Rhodophyta consisted of 32 families, 75 genera and 143 species (60% of all taxa) in the Costa das Algas EPA and 29 families, 49 genera and 68 species (53% of all taxa) in the Santa Cruz WR. Chlorophyta consisted of 15 families, 21 genera and 57 species (24% of all taxa) in the Costa das Algas EPA and 10 families, 14 genera and 30 species (22% of all taxa) in the Santa Cruz WR. Ochrophyta was composed of seven families, 16 genera and 39 species (16% of all taxa) in the Costa das Algas EPA and five families, 14 genera and 33 species (24% of all taxa) in the Santa Cruz WR. The most species-rich families were Rhodomelaceae (37 taxa) and Corallinaceae (11 taxa) amongst Rhodophyta; Caulerpaceae (13) and Cladophoraceae (13) amongst Chlorophyta and Dictyotaceae (21) and Sargassaceae (12) amongst Ochrophyta in both MPAs.

Nine species are Brazilian endemic: *Agisseainamoena*, *Alsidiumoliveiranum*, *Calliblepharisjolyi*, *Cryptonemiadelicatula*, *Hypneabrasiliensis*, *Laurencialongiramea*, *L.translucida* (Rhodophyta); *Laminariaabyssalis* (Ochrophyta) and *Halimedajolyana* (Chlorophyta) (Nunes 1998, Nunes 2005, Moura et al. 2015, Ximenes et al. 2017, Flora e Funga do Brasil 2023).

One hundred and four species (40% of all taxa), recorded before the 2015 themining disaster, were also presented in our 2018-2019 surveys. There were 86 new occurrences (34% of all taxa in the MPA) for the Costa das Algas EPA and 45 new ones (33% of all taxa in the MPA) for the Santa Cruz WR in the 2018-2019 surveys. On the other hand, 64 previously recorded taxa (45 Rhodophyta, 14 Chlorophyta and 5 Ochrophyta) were not found in our surveys.

Two hundred species were recorded during the four field surveys and eight sampling sites, however, 71 of which occurred only once or twice during sampling. These species were categorised as rare because they presented a very low frequency of occurrence in both MPAs. Conversely, only eight species (*Codiumintertextum*, *Anadyomenestellata*, *Dictyosphaeriaversluysii*, *Lobophoravariegata*, *Zonariatournefortii*, *Hydrolithonfarinosum*, *Ochtodessecundiramea* and *Dichotomariamarginata*) were conspicuous and were found at all eight sampling sites in all seasons.

## Discussion

Rhodophyta comprised 70% of the species recorded in previous inventory in the Espirito Santo State, which were carried out between 1969 and 2004, while Chlorophyta and Ochrophyta, class Phaeophyceae, comprised 15% (Guimarães 2003). Proportions recorded herein, from surveys carried out between 2018 and 2019, are slightly different once about 60% of the species recorded in this latter surveys belong to Rhodophyta. While Rhodophyta encompasses a high proportion of small filamentous and delicate species and tends to present higher richeness in areas with better environmental quality, Chlorophyta are smaller and filamentous forms, that tends to be more tolerant to changes in the environment [WFD-UKTAG (Water Framework Directive - United Kingdom Technical Advisory Group) 2009]. The red algal order, Ceramiales, which tends to thrive under better environmental quality (Bermejo et al. 2012) encompassed the higher richness in the study area (66 species), both before and after the 2015 mining disaster, but 22 of the previously recorded species of this group were not found during the latter surveys. An inventory carried out in the 2000s (Guimarães 2003), compared the Ceramiales flora of Espírito Santo (115 species) with surveys carried ou 30 years earlier (Oliveira-Filho 1969) and recorded no differences in the algal composition. Ecological impacts of terrestrial nutrients, sediments and pollutants are likely to be species-specific (Díaz-Pulido et al. 2007), and may lead to insidious changes in the macroalgal assemblages. The two protected areas studied herein are historically degraded due to multiple chronic impacts (Costa et al. 2021, Scherner et al. 2013), but the contaminants from the 2015 mining disaster are especially concerning and deserve further monitoring efforts (Quaresma et al. 2021, Coppo et al. 2023).

New records of algae after the mining disaster are due either to the increased sampling effort near the Doce river mouth or to taxonomic studies during the two last decades. Some of these newly recorded taxa are indeed recent description, and it should be also mentioned that amongst the 100 new records comprise rare species. Furthermore, there are new records amongst Dictyotales, Fucales and Corallinales, which are the most abundant algal groups and have an intricate taxonomy due to morphological variability associated to environmental conditions (Bahia 2014, González-Nieto et al. 2020). For example, in a recent molecular study, 10 species of *Sargassum* (*S.bermudense*, *S.buxifolium*, *S.cymosum*, *S.filipendula*, *S.fluitans*, *S.furcatum*, *S.hystrix*, *S.natans*, *S.polyceratium* and *S.vulgare*) were synonymised under *S.cymosum* (González-Nieto et al. 2020). Here, we did not use González-Nieto et al. (2020) taxonomic arrangement, as their analyses were largely based on data from the Caribbean with only one sequence from Brazil.

Our new algal records do not mean environmental quality improvements in the Espirito Santo coast. Indeed, new algal records are frequent in degraded environments, which tend to receive adequate monitoring efforts after enviroments impacts. Examples include Santos (Oliveira and Qi 2003), Sepetiba (Amado-Filho et al. 2003) and Guanabara bays (De Paula et al. 2020), as well as the vicinity of other urban centres (Scherner et al. 2013). Therefore, actual decreases in diversity are frequent despite larger checklists. For instance, Amado-Filho et al. (2003) recorded reduction of Rhodophyta richness, together with dominance of *Sargassum* spp. and *Padinagymnospora* under high metal concentrations in Sepetiba Bay (Brazil). Algal assemblages are excellent indicators of climate and anthropogenic impacts (Mazzucco et al. 2020), Carneiro et al. 2023).

The Costa das Algas Environment Protection Area (EPA) and Santa Cruz Wildlife Refuge (WR) are marine biodiversity hotspots threatened by mining wastes from the 2015 disaster, as well as by uncontrolled tourism, shrimp trawling, irregular discharge of chemicals, oils and solid wastes., invasive species and climate changes [ICMBio (Instituto Chico Mendes de Conservação da Biodiversidade) 2023b]. Long-term monitoring of these highly relevant and threatened MPAs is essential to guide management actions and restoration efforts, and the baseline data herein providesa relevant foundation for further assessments.

## Supplementary Material

XML Treatment for Chlorophyta/Ochrophyta/Rhodophyta/Tracheophyta

F94E1D0E-D138-5A30-A7A8-A5934D4F300D 10.3897/BDJ.12.e122350.suppl1 Supplementary material 1 Checklist of Data type Excel - taxonomy Brief description Checklist of marine macroalgae of the Costa das Algas Environmental Protected Area. File: oo_996209.xlsx https://binary.pensoft.net/file/996209 Authors: Claudia S. Karez, Ricardo G. Bahia, José M.C. Nunes, Gabriel N. Santos, Rodrigo L. Moura, Paulo S. Salomon, Clarice C. M. Ribeiro, Carolina S. Silva, Pedro Cardial, Gabriella A. Leal, Manoela B. Lyra, Leonardo T. Salgado

156E1A55-29EC-5B3B-A04A-565048700EEC 10.3897/BDJ.12.e122350.suppl2 Supplementary material 2 Checklist of Data type Excel - Taxonomy Brief description Checklist of marine macroalgae of the Santa Cruz Wildlife Refuge. File: oo_996215.xlsx https://binary.pensoft.net/file/996215 Authors: Claudia S. Karez, Ricardo G. Bahia, José M.C. Nunes, Gabriel N. Santos, Rodrigo L. Moura, Paulo S. Salomon, Clarice C. M. Ribeiro, Carolina S. Silva, Pedro Cardial, Gabriella A. Leal, Manoela B. Lyra, Leonardo T. Salgado

## Figures and Tables

**Table 1.** List of macroalgae of the Costa das Algas EPA (CA) and the Santa Cruz WR (SC) recorded in the present study (2018-2019 survey) and previous period (1969-2004). R = Record (R = taxa present in both periods: before 2004 and in this study (2018-2019); 1st R = first record to MPAs in this study (2018-2019); PR = taxa present only before 2004); A = Abundance (Ra = Rare; F = Frequent; C = Common; VC = Very common; Blank = unknown).

**PHYLUM/CLASS/ORDER/FAMILY/SPECIES**	**Costa das Algas EPA**	**Santa Cruz WR**	**Period record**	**Abundance**
**CHLOROPHYTA**				
**CLASS ULVOPHYCEAE**				
**BRYOPSIDALES**				
**Bryopsidaceae**				
*Bryopsispennata* J.V.Lamouroux 1809	X	X	PR	
*Bryopsisplumosa* (Hudson) C.Agardh 1823	X		PR	
**Caulerpaceae**				
*Caulerpaambigua* Okamura 1897	X		1st R	Ra
*Caulerpachemnitzia* (Esper) J.V.Lamouroux 1809	X	X	R	F
*Caulerpacupressoides* (Vahl) C.Agardh 1817	X	X	R	F
Caulerpacupressoidesvar.lycopodium Weber Bosse 1898	X		R	F
Caulerpacupressoidesvar.mamillosa (Montagne) Weber Bosse 1898	X		PR	F
*Caulerpafastigiata* Montagne 1837	X		R	F
*Caulerpalanuginosa* J.Agardh 1873	X	X	R	F
*Caulerpamexicana* Sonder ex Kützing 1849	X	X	R	F
*Caulerpaprolifera* (Forsskål) J.V.Lamouroux 1809	X	X	R	F
*Caulerparacemosa* (Forsskål) J.Agardh 1873	X	X	R	C
*Caulerpasertularioides* (S.G.Gmelin) M.Howe 1905	X	X	R	F
*Caulerpaverticillata* J.Agardh 1847	X		1st R	F
*Caulerpawebbian*a Montagne 1837	X		1st R	Ra
**Codiaceae**				
*Codiumdecorticatum* (Woodward) M.A.Howe 1911	X		R	
*Codiumintertextum* Collins & Hervey 1917	X	X	R	VC
*Codiumisthmocladum* Vickers 1905	X	X	R	C
*Codiumspongiosum* Harvey 1855	X		PR	
*Codiumtaylorii* P.C.Silva 1960	X	X	R	F
**Derbesiaceae**				
*Derbesiamarina* (Lyngbye) Solier 1846	X		1st R	Ra
**Dichotomosiphonaceae**				
*Avrainvillealongicaulis* (Kützing) G.Murray & Boodle 1889	X		PR	
*Avrainvilleanigricans* Decaisne 1842	X		1st R	Ra
**Halimedaceae**				
*Halimedadiscoidea* Decaisne 1842	X		1st R	Ra
*Halimedajolyana* Ximenes, Bandeira-Pedrosa, Cassano, Oliveira-Carvalho, Verbruggen & S.M.B.Pereira 2017	X	X	R	C
*Penicilluscapitatus* Lamarck 1813	X		1st R	Ra
**CLADOPHORALES**				
**Anadyomenaceae**				
*Anadyomenestellata* (Wulfen) C.Agardh 1823	X	X	R	VC
*Anadyomenerhizoidifera* A.B.Joly & S.Pereira 1973	X		R	
*Microdictyonboergesenii* Setchell 1925		X	R	
**Boodleaceae**				
*Boodleastruveoides* M.Howe 1918	X	X	1st R	Ra
*Cladophoropsismembranacea* Børgesen 1905	X	X	R	F
*Phyllodictyonanastomosans* (Harvey) Kraft & M.J.Wynne 1996	X		1st R	
**Cladophoraceae**				
*Chaetomorphaaerea* (Dillwyn) Kützing 1849	X	X	R	
*Chaetomorphaantennina* (Bory) Kützing 1847	X		1st R	Ra
*Chaetomorphabrachygona* Harvey 1858	X		1st R	Ra
*Chaetomorphalinum* (O.F.Müller) Kützing 1843	X		1st R	
*Chaetomorphaminima* Collins & Hervey 1917	X		1st R	Ra
*Chaetomorphanodosa* Kützing 1849	X		PR	
*Cladophoracoelothrix* Kützing 1843	X		R	Ra
*Cladophoracorallicola* Børgesen 1913	X	X	PR	
*Cladophoralaetevirens* (Dillwyn) Kützing 1843	X	X	1st R	F
*Cladophoraprolifera* (Roth) Kützing 1843	X	X	R	F
*Cladophorarupestris* (Linnaeus) Kützing 1843	X		PR	
*Cladophoravagabunda* (Linnaeus) Hoek 1963	X	X	R	Ra
*Willeellaordinata* Børgesen 1930	X	X	R	Ra
**Siphonocladaceae** F.Schmitz				
*Dictyosphaeriacavernosa* (Forsskål) Børgesen 1932	X	X	R	
*Dictyosphaeriaversluysii* Weber Bosse 1905	X	X	R	VC
*Siphonocladustropicus* (P.Crouan & H.Crouan) J.Agardh 1887	X		PR	
**Valoniaceae**				
*Valoniaaegagropila* C.Agardh 1823	X	X	R	F
*Valoniamacrophysa* Kützing 1843	X	X	R	F
*Valoniautricularis* (Roth) C.Agardh 1823	X		PR	
**DASYCLADALES**				
**Polyphysaceae**				
*Parvocaulismyriosporus* C.W.Nascimento Moura & J.C.DeAndrade 2014	X		1st R	Ra
**ULOTRICHALES**				
**Gayraliaceae**				
*Gayraliaoxysperma* (Kützing) K.L.Vinogradova ex Scagel & al. 1989	X		PR	
**ULVALES**				
**Ulvaceae**				
*Ulvaclathrata* (Roth) C.Agardh 1811	X		PR	
*Ulvaflexuosa* Wulfen 1803	X	X	R	F
*Ulvalactuca* Linnaeus 1753	X	X	R	Ra
*Ulvalinza* Linnaeus 1753	X	X	R	
*Ulvarigida* C.Agardh 1823	X	X	R	F
**Ulvellaceae**				
*Ulvellascutata* R.Nielsen, C.J.O'Kelly & B.Wysor 2013	X		PR	
*Ulvellaviridis* (Reinke) R.Nielsen, C.J.O'Kelly & B.Wysor 2013	X		PR	
Total	59	30		
**OCHROPHYTA**				
**CLASS PHAEOPHYCEAE**				
**DICTYOTALES**				
**Dictyotaceae**				
*Canistrocarpuscervicornis* (Kützing) De Paula & De Clerck 2006	X	X	R	C
*Canistrocarpuscrispatus* (J.V.Lamouroux) De Paula & De Clerck 2006	X	X	1st R	Ra
*Dictyopterisdelicatula* J.V.Lamouroux 1809	X	X	R	F
*Dictyopterisplagiogramma* (Montagne) Möbius 1889	X	X	R	F
*Dictyopterispolypodioides* (De Candolle) J.V.Lamouroux 1809	X	X	R	F
*Dictyotacaribaea* Hörnig & Schnetter 1992	X		1st R	Ra
*Dictyotaciliolata* Sonder ex Kützing 1859	X	X	R	F
*Dictyotacrenulata* J.Agardh 1847	X	X	1st R	F
*Dictyotadichotoma* (Hudson) J.V.Lamouroux 1809	X		PR	
*Dictyotajamaicensis* W.R.Taylor 1960	X	X	R	F
*Dictyotamenstrualis* Schnetter, Hörning & Weber-Peukert 1987	X	X	R	F
*Dictyotamertensii* (C.Martius) Kützing 1859	X	X	R	F
*Lobophoravariegata* (J.V.Lamouroux) Womersley ex E.C.Oliveira 1977	X	X	R	VC
*Padinaantillarum* (Kützing) Piccone 1886	X	X	R	F
*Padinaboergesenii* Allender & Kraft 1983		X	1st R	F
*Padinagymnospora* (Kützing) Sonder 1871	X	X	R	F
*Padinasanctae-crucis* Børgesen 1914	X		1st R	F
*Spatoglossumschroederi* (C.Agardh) Kützing 1859	X	X	R	C
*Stypopodiumzonale* (J.V.Lamouroux) Papenfuss 1940	X	X	PR	
*Zonariatournefortii* (J.V.Lamouroux) Montagne 1846	X	X	R	VC
*Zonariazonata* C.Agardh	X	X	1st R	Ra
**ECTOCARPALES**				
**Acinetosporaceae**				
*Acinetosporafilamentosa* (Noda) Yaegashi, Uwai & Kogame 2015		X	1st R	Ra
*Feldmanniairregularis* (Kützing) Hamel 1939	X	X	1st R	Ra
*Feldmanniamitchelliae* (Harvey) H.-S.Kim 2010		X	1st R	Ra
**Scytosiphonaceae**				
*Chnoosporaminima* (Hering) Papenfuss 1956	X		1st R	Ra
*Colpomeniasinuosa* (Mertens ex Roth) Derbès & Solier 1851	X	X	R	C
*Hydroclathrusclathratus* (C.Agardh) M.Howe 1920	X	X	R	F
**FUCALES**				
**Sargassaceae**				
*Sargassumcymosum* C.Agardh 1820	X		1st R	Ra
Sargassumcymosumvar.nanum E.de Paula & E.C.Oliveira	X		1st R	
*Sargassumfilipendula* C.Agardh 1824	X	X	R	F
*Sargassumfurcatum* Kützing 1843	X	X	R	Ra
*Sargassumhystrix* J.Agardh 1847	X	X	R	F
*Sargassumnovae-hollandiae* P.C.Silva 1996	X	X	1st R	F
*Sargassumplatycarpum* Montagne 1842	X		PR	
*Sargassumpolyceratium* Montagne 1837	X	X	1st R	F
*Sargassumramifolium* Montagne 1843	X	X	R	F
*Sargassumrigidulum* Kützing 1849	X	X	1st R	F
*Sargassumstenophyllum* C.Martius 1828	X	X	R	F
*Sargassumvulgare* C.Agardh 1820	X	X	1st R	F
**LAMINARIALES**				
**Laminariaceae**				
*Laminariaabyssalis* A.B.Joly & E.C.Oliveira 1967	X		PR	
**SCYTOTHAMNALES**				
**Bachelotiaceae**				
*Bachelotiaantillarum* (Grunow) Gerloff 1959	X		1st R	Ra
**SPHACELARIALES**				
**Sphacelariaceae**				
*Sphacelariabrachygonia* Montagne 1843	X		PR	
*Sphacelariarigidula* Kützing 1843	X	X	R	F
Total	40	33		
**RHODOPHYTA**				
**CLASS FLORIDEOPHYCEAE**				
**CERAMIALES**				
**Callithamniaceae**				
*Aglaothamnionfelipponei* (Howe) Aponte, Ballantine & J.N.Norris 1994	X		PR	
*Aglaothamnionherveyi* (M.Howe) N.E.Aponte, D.L.Ballantine, & J.N.Norris 1994	X		1st R	Ra
*Spyridiaaculeata* (C.Agardh ex Decaisne) Kützing 1843	X		PR	
*Spyridiaclavata* Kützing 1841		X	1st R	Ra
*Spyridiafilamentosa* (Wulfen) Harvey 1833	X		1st R	Ra
*Spyridiahypnoides* (Bory) Papenfuss 1968	X		PR	
**Ceramiaceae**				
*Centrocerasgasparrinii* (Meneghini) Kützing 1849	X	X	1st R	F
*Ceramiumluetzelburgii* O.C.Schmidt 1924	X	X	1st R	Ra
*Ceramothamnionbrasiliensis* (A.B.Joly) M.J.Wynne & C.W.Schneider 2023	X		PR	
*Ceramothamnioncodii* H.Richards 1901		X	1st R	Ra
*Gaylielladawsonii* (A.B.Joly) Barros-Barreto & F.P.Gomes 2020	X		1st R	F
*Pseudoceramiumcaraibicum* (H.E.Petersen & Børgesen) Barros-Barreto, Maggs & M.A.Jaramillo 2023	X		1st R	Ra
**Delesseriaceae**				
*Acrosoriumciliolatum* (Harvey) Kylin 1924	X		1st R	Ra
*Caloglossaleprieurii* (Montagne) G.Martens1869	X		PR	
*Cryptopleuracrispa* Kylin 1924	X		PR	
*Cryptopleuraramosa* (Hudson) L.Newton 1931	X		1st R	Ra
*Dasyacorymbifera* J.Agardh 1841	X		R	Ra
*Dasyarigidula* (Kützing) Ardissone 1878	X		1st R	Ra
*Dictyurusoccidentalis* J.Agardh 1847	X		1st R	Ra
*Heterosiphoniacrispella* (C.Agardh) M.J.Wynne 1985	X	X	R	Ra
*Heterosiphoniagibbesii* (Harvey) Falkenberg 1901	X	X	R	F
*Taeniomaperpusillum* (J.Agardh) J.Agardh 1863	X		1st R	Ra
*Thuretiabornetii* Vickers 1905		X	1st R	Ra
**Rhodomelaceae**				
*Acanthophoraspicifera* (M.Vahl) Børgesen 1910	X	X	R	F
*Alsidiumoliveiranum* S.M.Guimarães & M.T.Fujii 2019	X		PR	
*Alsidiumseaforthii* (Turner) J.Agardh 1841	X	X	R	C
*Bostrychiabinderi* Harvey 1849	X	X	1st R	F
*Bostrychiamontagnei* Harvey 1853	X		PR	
*Bostrychiaradicans* (Montagne) Montagne 1842	X		PR	
*Bostrychiascorpioides* (Hudson) Montagne 1842	X		PR	
*Bostrychiatenella* (J.V.Lamouroux) J.Agardh 1863	X		R	Ra
*Bryocladiacuspidata* (J.Agardh) De Toni 1903	X	X	R	
*Carradorielladenudata* (Dillwyn) Savoie & G.W.Saunders 2019	X	X	1st R	Ra
*Chondriacapillaris* (Hudson) M.J.Wynne 1991	X		PR	
*Chondrialittoralis* Harvey 1853	X		PR	
*Chondriasedifolia* Harvey 1853	X		PR	
*Dipterosiphoniadendritica* (C.Agardh) F.Schmitz 1897	X	X	1st R	Ra
*Herposiphonianuda* Hollenberg 1968	X	X	1st R	Ra
*Herposiphoniasecunda* (C.Agardh) Ambronn 1880	X	X	R	
*Herposiphoniatenella* (C.Agardh) Ambronn 1880	X		R	Ra
*Laurenciaaldingensis* Y.Saito & Womersley 1974	X		PR	
*Laurenciaarbuscula* Sonder 1845	X		1st R	F
*Laurenciadendroidea* J.Agardh 1852	X	X	R	F
*Laurenciacatarinensis* Cordeiro-Marino & Fujii 1985	X		PR	
*Laurenciaintricata* J.V.Lamouroux 1813		X	1st R	
*Laurenciafiliformis* (C.Agardh) Montagne 1845	X		PR	
*Laurencialongiramea* Cassano, G.N.Santos, J.M.C.Nunes, M.C.Oliveira & M.T.Fujii 2019	X		PR	
*Laurenciaoliveirana* Yoneshigue-Valetin, M.J.Wynne & Cassano 2022	X		R	Ra
*Laurenciatranslucida* Fujii & Cordeiro-Marino 1996	X		PR	
*Laurenciavenusta* Yamada 1931	X		PR	
*Melanothamnusferulaceus* (Suhr ex J.Agardh) Díaz-Tapia & Maggs 2017	X		1st R	F
*Melanothamnusgorgoniae* (Harvey) Díaz-Tapia & Maggs 2017		X	1st R	Ra
*Melanothamnustongatensis* (Harvey ex Kützing) Díaz-Tapia & Maggs 2017		X	R	Ra
*Osmundariaobtusiloba* (C.Agardh) R.E.Norris 1991	X	X	R	C
*Palisadacorallopsis* (Montagne) Sentíes, Fujii & Díaz-Larrea 2008	X		1st R	Ra
*Palisadaflagellifera* (J.Agardh) K.W.Nam 2007	X		PR	
*Palisadafurcata* (Cordeiro-Marino & M.T.Fujii) Cassano & M.T.Fujii 2012	X	X	R	F
*Palisadaperforata* (Bory) K.W.Nam 2007	X	X	R	F
*Polysiphoniasertularioides* (Grateloup) J.Agardh 1863	X	X	1st R	F
*Polysiphoniasubtilissima* Montagne 1840		X	R	Ra
**Wrangeliaceae**				
*Anotrichiumelongatum* (Harvey) Baldock 1976	X	X	PR	
*Griffithsiaglobulifera* Harvey ex Kützing 1862		X	1st R	Ra
*Griffithsiaschousboei* Montagne 1841	X	X	1st R	F
*Haloplegmaduperreyi* Montagne 1842	X		1st R	
*Wrangeliaargus* (Montagne) Montagne 1856	X		R	F
*Wrangeliapenicillata* (C.Agardh) C.Agardh 1828	X		PR	
**CORALLINALES**				
**Corallinaceae**				
*Arthrocardiaflabellata* (Kützing) Manza 1940	X		PR	
*Arthrocardiavariabilis* (Harvey) Weber Bosse 1904	X		1st R	
*Corallinaofficinalis* Linnaeus 1758	X		R	F
*Corallinapanizzoi* R.Schnetter & U.Richter 1979	X	X	R	F
*Janiacapillacea* Harvey 1853	X		PR	
*Janiacrassa* J.V.Lamouroux 1821	X		PR	
*Janiacubensis* Montagne ex Kützing 1849	X	X	1st R	F
Janiapedunculatavar.adhaerens (J.V.Lamouroux) A.S.Harvey, Woelkerling & Reviers 2020	X		1st R	F
*Janiarubens* (Linnaeus) J.V.Lamouroux 1816	X		R	Ra
*Janiasagittata* (J.V.Lamouroux) Blainville 1834	X		PR	
*Janiasubulata* (Ellis & Solander) Sonder 1848	X		R	Ra
**Hydrolithaceae**				
*Hydrolithonfarinosum* (J.V.Lamouroux) Penrose & Y.M.Chamberlain 1993	X	X	1st R	VC
*Hydrolithon* sp. (Foslie) Foslie, 1909	X	X	PR	
**Lithophyllaceae**				
*Amphiroaanastomosans* Weber Bosse 1904	X	X	R	C
*Amphiroabeauvoisii* J.V.Lamouroux 1816	X		PR	
*Amphiroabrasiliana* Decaisne 1842	X		1st R	Ra
*Amphiroafragilissima* (Linnaeus) J.V.Lamouroux 1816	X	X	R	F
*Amphiroarigida* J.V.Lamouroux 1816	X	X	R	F
*Lithophyllumcorallinae* (P.Crouan & H.Crouan) Heydrich 1897		X	1st R	F
*Lithophyllumprototypum* (Foslie) Foslie 1905	X		1st R	F
*Lithophyllum* sp. Philippi, 1837	X	X	1st R	F
**Porolithaceae**				
*Harveylithonroseum* C.Liu & S.-M.Lin 2018	X	X	1st R	F
**Spongitidaceae**				
*Neogoniolithonbrassica-florida* (Harvey) Setchell & L.R.Mason 1843	X		1st R	F
**GELIDIALES**				
**Gelidiaceae**				
*Gelidiumamericanum* (W.R.Taylor) Santelices 1976	X		PR	
*Gelidiumcapense* (S.G.Gmelin) P.C.Silva 1987	X	X	R	F
*Gelidiumfloridanum* W.R.Taylor 1943	X	X	R	F
*Gelidiumpusillum* (Stackhouse) Le Jolis 1863	X		PR	
*Gelidiumtorulosum* Kützing 1868	X		PR	
**Gelidiellaceae**				
*Gelidiellaacerosa* (Forsskål) Feldmann & Hamel 1934	X	X	R	C
Gelidiella ligulata E.Y.Dawson 1953	X		1st R	Ra
*Parviphycustrinitatensis* (W.R.Taylor) M.J.Wynne 2010	X		1st R	Ra
**Pterocladiaceae**				
*Pterocladiellabartlettii* (W.R.Taylor) Santelices 1998	X	X	R	F
*Pterocladiellabeachiae* Freshwater 2001	X	X	1st R	F
*Pterocladiellacapillacea* (S.G.Gmelin) Santelices & Hommersand 1997	X		R	Ra
**GIGARTINALES**				
**Caulacanthaceae**				
*Catenellacaespitosa* (Withering) L.M.Irvine 1976	X	X	R	F
**Cystocloniaceae**				
*Calliblepharisjolyi* E.C.Oliveira 1970	X		PR	
*Hypneabrasiliensis* P.B.Jesus, Nauer & J.M.C.Nunes 2016	X	X	1st R	F
*Hypneacervicornis* J.Agardh 1851	X	X	R	F
*Hypneapseudomusciformis* Nauer, Cassano & M.C.Oliveira 2015	X	X	R	C
**Gigartinaceae**				
*Chondracanthusacicularis* (Roth) Fredericq 1993	X	X	R	F
*Chondracanthusteedei* (Mertens ex Roth) Kützing 1843	X		PR	
**Phyllophoraceae**				
*Gymnogongrusgriffithsiae* (Turner) C.Martius 1833	X		R	Ra
*Petroglossumundulatum* C.W.Schneider 1976	X	X	R	Ra
**Rhizophyllidaceae**				
*Ochtodessecundiramea* (Montagne) M.Howe 1920	X	X	1st R	VC
**Solieriaceae**				
*Meristothecagelidium* (J.Agardh) E.J.Faye & M.Masuda 2004	X		R	Ra
*Solieriafiliformis* (Kützing) Gabrielson 1985	X	X	R	F
**GRACILARIALES**				
**Gracilariaceae**				
*Gracilariacaudata* J.Agardh 1852	X		PR	
*Gracilariacearensis* (A.B.Joly & Pinheiro) A.B.Joly & Pinheiro 1966	X	X	1st R	Ra
*Gracilariacervicornis* (Turner) J.Agardh 1852	X	X	R	F
*Gracilariacuneata* Areschoug 1854	X	X	R	Ra
*Gracilariadomingensis* (Kützing) Sonder ex Dickie 1874	X	X	1st R	F
*Gracilariaferox* J.Agardh 1852	X		1st R	
*Gracilariaflabelliformis* (P.Crouan & H.Crouan) Fredericq & Gurgel 2004	X		1st R	
Gracilariaflabelliformissubsp.simplex Gurgel, Fredericq & J.N.Norris 2004	X		1st R	Ra
*Gracilariafoliifera* (Forsskål) Børgesen 1932	X		1st R	
*Gracilariamammillaris* (Montagne) M.Howe 1918	X		PR	
*Gracilariarangiferina* (Kützing) Piccone 1886	X		1st R	Ra
**HALYMELIALES**				
**Grateloupiaceae**				
*Grateloupiafilicina* (J.V.Lamouroux) C.Agardh 1822	X	X	R	Ra
*Grateloupiafiliformis* Kützing 1849	X		PR	
**Halymeniaceae**				
*Cryptonemiabermudensis* Collins & M.Howe) C.W.Schneider, C.E.Lane & G.W.Saunders 2018	X		PR	
*Cryptonemiabengryi* W.R.Taylor 1960	X		PR	
*Cryptonemiadelicatula* Joly & Cordeiro 1966	X		PR	
*Cryptonemiaseminervis* (C.Agardh) J.Agardh 1846	X	X	R	F
*Halymeniabrasiliana* S.M.P.B.Guimarães & M.T.Fujii 1998	X		1st R	Ra
*Halymeniaelongata* C.Agardh 1822	X		PR	
*Halymeniafloridana* J.Agardh 1892	X		1st R	Ra
**HAPALIDIALES**				
**Hapalidiaceae**				
*Lithothamnionmuelleri* Lenormand ex Rosanoff 1866	X		PR	
*Phymatolithon* sp. Foslie, 1898, nom. cons.	X		1st R	F
*Roseolithoncrispatum* (Hauck) P.W.Gabrielson, Maneveldt, Hughey & V.Peña 2023	X	X	R	C
*Roseolithon* sp. L.M.Coutinho & Barros-Barreto, 2021	X	X	1st R	
**Mesophyllumaceae**				
*Mesophyllum* sp. Me.Lemoine, 1928	X		1st R	F
**NEMALIALES**				
**Galaxauraceae**				
Dichotomariamarginata (J.Ellis & Solander) Lamarck 1816	X	X	R	VC
Dichotomaria obtusata (J.Ellis & Solander) Lamarck 1816	X	X	R	F
Galaxaura rugosa (J.Ellis & Solander) J.V.Lamouroux 1816	X	X	1st R	Ra
Tricleocarpacylindrica (J.Ellis & Solander) Huisman & Borowitzka 1990	X	X	R	F
Tricleocarpa fragilis (Linnaeus) Huisman & R.A.Townsend 1993	X	X	1st R	C
**Liagoraceae**				
*Gloiocallisdendroidea* (P.Crouan & H.Crouan) Showe M.Lin, Huisman & D.L.Ballantine 2014	X		1st R	Ra
*Helminthocladiacalvadosii* (J.V.Lamouroux ex Duby) 1915	X		PR	
*Liagoraceranoides* J.V.Lamouroux 1816	X		1st R	Ra
**Scinaiaceae**				
*Scinaiahalliae* (Setchell) Huisman 1985	X		R	F
**PEYSSONNELIALES**				
**Peyssonneliaceae**				
*Agisseainamoena* (Pilger) Pestana, Lyra, Cassano & J.M.C. Nunes 2021	X		PR	
*Peyssonneliaarmorica* (P.Crouan & H.Crouan) Weber Bosse 1916	X		PR	
*Peyssonneliaboergesenii* (S.G.Gmelin) Decaisne ex J.Agardh 1916	X		PR	
*Peyssonnelia* sp. Decaisne, 1841	X	X	R	C
**PLOCAMIALES**				
**Plocamiaceae**				
*Plocamiumbrasiliense* (Greville) M.Howe & W.R.Taylor 1931	X	X	R	F
**RHODYMENIALES**				
**Champiaceae**				
*Champiafeldmannii* Díaz-Piferrer 1977	X	X	R	F
*Champiavieillardii* Kützing 1866	X	X	1st R	F
**Lomentariaceae**				
*Ceratodictyonplanicaule* (W.R.Taylor) M.J.Wynne 2011	X	X	1st R	Ra
*Ceratodictyonvariabile* (J.Agardh) R.E.Norris 1987	X	X	1st R	F
**Rhodymeniaceae**				
*Botryocladiaoccidentalis* (Børgesen) Kylin 1931	X	X	R	Ra
*Botryocladiawynnei* D.L.Ballantinne 1985	X	X	1st R	F
*Rhodymeniadivaricata* E.Y.Dawson 1941	X		R	
*Rhodymeniapseudopalmata* (J.V.Lamouroux) P.C.Silva 1952	X	X	R	F
**SPOROLITHALES**				
**Sporolithaceae**				
*Sporolithonepisporum* (M.Howe) E.Y.Dawson 1960	X		1st R	F
*Sporolithon* sp. Heydrich, 1897		X	1st R	F
Total	150	73		
TOTAL	249	136		
**TRACHEOPHYTA**				
**CLASS MONOCOTS**				
**ALISMATALES**				
**Cymodoceaceae**				
*Halodulewrightii* Ascherson 1868	X	X	1st R	
